# 

**Published:** 1889-07-13

**Authors:** 


SATURDAY, JULY 13th, 1889.

**A Physiological Study** - - - - - - - - 223
**Editor's Letter-Box.**—The "Doctor's" Dictum Questioned 224
**Mrs. Gladstone's Home in Epping Forest** - - - - 225
**Dr. Richardson, F.R.S., as a Novelist** - - - - 226
**The Doctor's Pulpit.**—In Nature's School - - - - 227
**Within the Wards.**—Is "High" Game Poisonous?—A Steak-pie Story - - - - - - - - - - - 228
**The Story of the Insane from Year to Year.**—Asylum Reports - - - - - - - - - - 229
**Notes and News** - - - - - - - - - - - 230
**Everybody's Page** - - - - - - - - - - 232
**Annotations.**—"Voiceless Wives"—Convocation and Gambling—Pure Air for Dyspeptics - - - - - 233
**The Hospital Workshop.**—No. V. The National Hospital for the Paralysed and Epileptic - - - - - 234
**Her Heart's Mistake.** By E. D. Cumming. - - - 235
**What Will Trained Nurses Gain by Joining the British Nurses' Association?** - - - - - 237
**Children's Country Holiday Fund** - - - - - 238
**Amusements—Scraps and Gleanings** - - - - 238

**EXTRA SUPPLEMENT—THE NURSING MIRROR.**

**En Passant.**—The Teaching of Nurses—Church Army Nurses—Salisbury—Summer Afternoons—Florence Nightingale—Cooking for Invalids—Orkney—Institutes at Bath - - - - - - - - - - - - lv
**Physiology for Nurses.**—No. 1. The Skin- - - - lvi
**The National Pension Fund.**—The Princess of Wales becomes President—A Bonus Fund of £26,000 - - - lvii
**Everybody's Opinion.**—Registration—Troubles at Birmingham—A Plea for Nurses - - - - - - -lviii
**Vacancies—Notes and Queries** - - - - - -lviii

